# Effects of changes in living environment on physical health: a prospective German cohort study of non-movers

**DOI:** 10.1093/eurpub/ckz044

**Published:** 2019-03-18

**Authors:** Benjamin Aretz, Gabriele Doblhammer, Fanny Janssen

**Affiliations:** 1 Institute of Sociology and Demography, University of Rostock, Rostock, Germany; 2 Department of Demography, Faculty of Spatial Sciences, University of Groningen, Groningen, The Netherlands; 3 German Centre for Neurodegenerative Diseases, Bonn, Germany; 4 Interdisciplinary Demographic Institute, The Hague, The Netherlands

## Abstract

**Background:**

Longitudinal studies on associations between changes in living environment and health are few and focus on movers. Next to causal effects, differences in health can, however, result due to residential mobility. The present study explored changes in living environment related to (changes in) physical health among non-movers. Causality was reinforced by a novel study design.

**Methods:**

We obtained longitudinal data on both living environment and physical health covering 4601 non-movers aged 18+ with 16 076 health observations from the German Socio-Economic Panel between 1999 and 2014. Changing and stable perceived living environment from three domains (infrastructure, environmental pollution, housing conditions) were included at household level. We performed linear regressions with robust standard errors and generalized estimating equations to predict the physical component summary (PCS) at baseline and changes in PCS over time.

**Results:**

Stable moderate and worst as well as worsened environmental pollution and infrastructure were associated with worse PCS at baseline, as were stable poor and worsened housing conditions. Stable worst infrastructure was associated with negative changes in PCS for both sexes. Men’s changes in PCS were more affected by worsened environmental pollution than women’s.

**Conclusion:**

A suboptimal living environment has short- and long-term negative effects on physical health. Because even short-term changes in the living environment have an immediate influence on an individual’s health status and health trajectories, public attention to living environment is essential to fight existing health inequalities.

## Introduction

Numerous epidemiological studies have found that an advantaged living environment was associated with good health and a disadvantaged living environment with worse health.^1–6^ Accordingly, the living environment is an important dimension of public health; it strengthens social and health inequalities.

However, most previous studies on the topic have pursued cross-sectional designs^7^ (see Schüle and Bolte for a review) or just used the baseline measurement of living environment characteristics in a longitudinal design^8^ and cannot control for social selection.^9^^,^^10^ Other studies concentrated only on the movers^3^^,^^4^ but those approaches may lead to biased results due to specific individual characteristics that may affect the decision to move (e.g. health, socio-economic determinants)^11^ and they neglect secular changes in living environments of the non-movers.

The few previous longitudinal studies^3^^,^^4^^,^^6^ found less evidence supporting the hypothesis of causal environmental effects on people’s health, or found only weak evidence for the beneficial effects of advantaged living environments. One study identified lower mortality risks for people living in greener areas,^12^ but another study detected hardly any positive health effect of moving to a neighbourhood with more green qualities.^6^

The unique contribution of our study is that we explored longitudinal associations of changing or stable living environments characteristics related to physical health and most importantly, subsequent health changes among non-movers in Germany. We impose a strict time order between cause and outcome and control for time-varying individual characteristics. We hypothesized that disadvantaged or worsening living environments are associated with a negative health and health development over time; whereby beneficial or improving living environments may lead to good health and positive changes in physical health.

## Methods

### Data and sample

Longitudinal data from 1999 to 2014 were obtained from the publicly available German Socio-Economic Panel (GSOEP), a representative prospective cohort study of German adults.^13^ The yearly waves contain, among other information, data on socio-economic and sociodemographic characteristics at the individual level. Information on the living environment at the household level is available on a 5-year basis: 1999, 2004 and 2009. Physical health in the form of the physical component summary (PCS) (see outcomes) is available on a 2-year basis from 2002 onwards.

The present study used all participants aged 18 and older at baseline. The baseline is defined as the first health measurement of people in the age 18 or older from wave 2004 onwards and took place in the waves 2004, 2006, 2008, 2010 due to the 2-year basis of the health data. A minimum of two health measurements and two observations of the living environmental characteristics were required to become part of the analysis population (see Supplementary figure S3).

The final analysis population covered 4601 non-movers residing in Germany and aged 18 and older at baseline (in 2004, 2006, 2008, 2010) with a total of 16 076 health observations and 11 475 health changes (from 2004 to 2014). The total number of changes in PCS covers all changes in PCS within a person summed up over all participants. This study was conducted in accordance with all principles embodied in the Declaration of Helsinki.

### Study design

**Figure 1:**
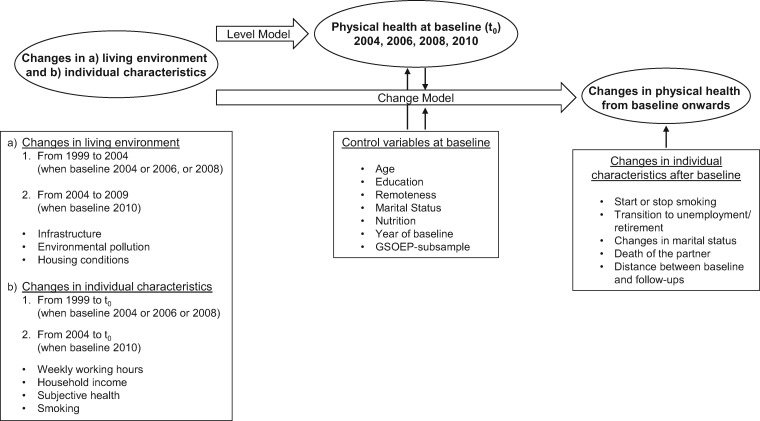
Study design for the analysis of changes in the living environment on physical health among German non-movers aged 18 and above

We strengthened the causal explanatory power of our findings by using a novel approach including four methodological strategies that reduce the confounding effects by selected migration/health selection into living environments:

(i) imposing a strict time order between living environment and physical health to exclude the possibility of reverse causation, (ii) predicting changes in health over time and not only in regard different health levels, (iii) including only non-movers, among whom health selection into living environments does not play a role and (iv) controlling for important time-invariant and time-varying individual characteristics. We defined two models: the Level Model and the Change Model. In the Level Model, we related the health status at baseline to changes in the environment and in individual characteristics before baseline. In the Change Model, we explored changes in health from baseline onwards, dependent on changes in the environment before baseline, as well as changes in individual characteristics before and after baseline, and health at baseline (figure 1). To ensure that the Change Model does not indicate participants’ migration trajectories through relocations in new living environments after baseline, we excluded after baseline movers to avoid potential confounding.

### Measures

#### Outcomes

Physical health was measured by the PCS, which is one of the two main dimensions of the 12-Item Short Form Survey version 2, invented by the RAND Corporation.^14^ PCS is a psychometric tool and consists of six self-reported variables (5-point Likert scale): two on physical functioning, one on general health, one on bodily pain and two on the role of functioning, which altogether loaded on one principal component, called PCS.^15^ The GSOEP reports the PCS as a metric variable (min = 0; max = 100) with higher scores indicating better health. The score was mean-centred to a value of 50, which means that scores lower or higher than 50 indicate worse or better health than the average in the whole GSOEP sample. For the baseline outcome (*n* = 4601), we estimated the reliability of PCS indicating a high internal consistency with a Cronbach’s alpha of 0.88.

In the Level Model, PCS is the main outcome measure. In the Change Model, a change in physical health ($\Delta y_{i}$) from baseline onwards is the main outcome measure. We used the baseline PCS score as a basis to calculate the change scores. A change $\Delta y_{i}=y_{\mathrm{it}}-\;y_{{\mathrm{it}}_{0}}$ is the difference between the PCS score from a following valid wave (t) of a subject (i) minus the PCS score of the baseline $\left(t_{0}\right)$. Thus negative scores of Δy  indicate individual health deterioration compared with baseline PCS score, a score of zero denotes unchanged health and positive scores individual health improvements. We used a maximum of three changes in PCS for one individual from baseline onwards to ensure reasonable proximity between measures of living environment and health.

#### Predictors

We included predictors from two main domains, namely the living environment which is our domain of interest, and individual characteristics which may confound our results. We captured three external dimensions of the living environment, namely infrastructure, environmental pollution and housing conditions, and, distinguished between stable, improved and worsened living environments. Additionally, we added relocation to identify only participants who did not move. Remoteness, which measured the distance of the people’s residence to the next city centre at baseline, served as a control variable. As for the individual characteristics, we identified relevant demographic, socio-economic and lifestyle determinants from the literature covering age, sex, education, weekly working hours, household income, smoking, marital status, death of the partner and subjective health. Table 1 provides the list of all abovementioned predictors, their full descriptions, the reclassifications and the final categories. In addition, we accounted for design variables: the year of baseline (at baseline), the GSOEP-subsample (at baseline) which indicates the random sample the participant belongs to,^13^ and the distance between the PCS follow-up to the baseline.

**Table 1:** Measures of time-invariant and time-varying living environment and individual characteristics, German Socio-Economic Panel 1999–2014

Time period^a^	Time dimension^b^	Domain	Measure	Description	Reclassification/calculation	Final categories
Up to baseline	Time-varying	Living environment	Infrastructure	Accessibility to retail, (social) services and public transport (11 items, 5-point Likert scale, Cronbach’s alpha = 0.98, all items loaded on one factor with eigenvalue > 1 estimated by principal component analysis)	Aggregation into an average Likert scale per wave with a minimum of five valid items to be included [range, 1–3.96]. Changes greater than 1 SD between the two measurements were coded as improved or worsened infrastructure. All others were allocated to stable infrastructure by forming the average score of the two measurements and dividing this into tertiles	Stable best, stable moderate, stable worst, improved, worsened
			Environmental pollution	Disturbances on air pollution, noise pollution and lack of green spaces (5-point Likert scale, Cronbach’s alpha = 0.74, all items loaded on one factor with eigenvalue > 1 estimated by principal component analysis)	Aggregation into one summary scale [range, 3–15]. Changes greater than 1 SD between the two measurements were coded as improved or worsened pollution. All others were allocated to stable pollution by forming the average score of the two measurements and dividing this into tertiles	Stable best, stable moderate, stable worst, improved, worsened
			Housing conditions	An item asking for inside conditions of the residential building	Aggregation of the two highest and the two lowest categories Moves of one category were coded as improved or worsened housing conditions	Stable good, stable in need of renovation, improved, worsened
			Relocation	A question since which year people live in actual residential building	Changes in the year of living in actual residential building	Yes (movers), no (non-movers)
		Individual characteristics	Weekly working hours	An item asking for weekly working hours	Aggregation of persons that were not employed, in vocational training, in military service, community service or worked in a sheltered workshop	Stable full-time employment, stable part-time employment, stable marginal employment, stable not employed/retired, increased working hours, decreased working hours
			Household income	An item asking for the yearly post-government household income	Dividing into income quintiles	Stable 1. quintile, stable 2. quintile, stable 3. quintile, stable 4. quintile, stable 5. quintile, more income, less income
			Subjective health	A question on how the person rated the own health in general	No reclassification applied	Stable very good, stable good, stable satisfactory, stable poor, stable bad, improved, worsened
			Smoking	A question about whether persons smoke	Aggregation of non-smokers and former smokers	Yes, no, started smoking, stopped smoking
At baseline	Time-invariant	Living environment	Remoteness	An item asking for the distance in kilometres to the next city centre	No reclassification applied	<10, 10–24, 25–39, 40–59, >59
		Individual characteristics	Age	A question on when the person was born	Difference between wave year and birth year	Metric variable ranged between 18 and 96
			Sex	An item asking for the sex	No reclassification applied	Male, female
			Education	An item asking for highest school degree	Aggregation of the ISCED-97 scale into three educational groups	Low, middle, high
			Marital status	An item asking for the person’s marital status	No reclassification applied	Married, single, widowed, divorced, separated
			Nutrition	A question about to what extent do persons follow a health-conscious diet	No reclassification applied	Very much, much, not so much, not at all
After baseline	Time-varying	Individual characteristics	Unemployment/retirement	Event/transition variable (dummy) that measures when persons became unemployed/retired	Comparison of the previous state at baseline and the state at waves afterwards	Unemployment/retirement (yes)
			Marital status	Event/transition variables (dummies) that measures when persons experienced changes in marital status	Comparison of the previous state at baseline and the state at waves afterwards	Married, single, widowed, divorced, separated (yes)
			Death of the partner	Event/transition variable (dummy) that measures when persons experienced a death of the partner	Comparison of the previous state at baseline and the state at waves afterwards	Death of the partner (yes)
			Start/stop Smoking	Event/transition variables (dummies) that measures when persons started or stopped smoking	Comparison of the previous state at baseline and the state at waves afterwards	Start smoking (yes)
						Stop smoking (yes)

Notes: ISCED-1997, International Standard Classification of Education 1997.

a Three different time periods were distinguished, namely the period up to baseline, the period at baseline and the period after baseline.

b Time dimension indicates whether the measures have time-invariant or time-varying values.

From both domains, living environment and individual characteristics, the predictors were included either as time-invariant variables (at baseline) or as time-varying ones (up to baseline/from baseline onwards).

All time-varying living environmental characteristics were calculated by forming the difference of the two available assessments. They were assessed by the key-person of the household (household head) and were then linked to all individuals in the same household.

All time-varying individual characteristics up to baseline were calculated by forming the difference between the measurement of each covariate at the time of first wave of living environment examination (1999 or 2004) and the assessment at baseline of this variable. For both individual and living environmental characteristics, we defined a change (for metric variables equal or greater than 1 SD) across all waves as improved or worsened living environment and distinguished between stable, improved and worsened characteristics.

In the Change Model, we added some event variables controlling for changes in individual characteristics after baseline. They were represented through several dichotomous variables, with the value one if an event occurred and zero otherwise.

### Statistical analysis

In the Level Model, we examined associations between changes in the living environment and in individual characteristics before or up to baseline and PCS at baseline using linear regressions. We selected the Level Model with the highest adjusted R squared and applied robust standard errors by Huber/White^16^^,^^17^ due to heteroscedastic residuals (Breusch-Pagan test: *P* < 0.001). In the Change Model, we performed generalized estimating equations^18^^,^^19^ using the identity link function and a normally distributed outcome variable (= changes in PCS score). By doing this, we controlled for multiple observations per person taking the autocorrelation of repeated measurements of the same persons into account. The within-person residual covariance matrix was specified by an independent correlation structure based on the quasi-likelihood information criterion.^20^ The Change Model with the best goodness of fit was identified by using the quasi-likelihood information criterion as well. All three living environment variables were included simultaneously in the Level and the Change Model. All calculations were performed using Stata/IC 12.1, and procedures ‘reg’ and ‘xtgee’.

## Results

The analysis sample consisted of 4601 participants, of whom 2171 (47.19%) were men and 2430 (52.81%) women. In this sample, 720 (15.6%) experienced changing infrastructure, 686 (14.91%) differences in environmental pollution and 873 (19.0%) changes in housing conditions (Supplementary table S3).

From baseline onwards, we included 16 076 PCS observations which resulted in 11 475 changes in PCS, of which 4980 were positive health changes and 6495 negative changes. PCS changes ranged between −46.24 and 40.46, with an average decline of −1.49 overall PCS changes and stronger average declines for women (−1.56) than men (−1.41) over time.

### Level Model

Changes in living environmental characteristics influenced health at baseline (table 2) compared with those experiencing stable best characteristics. People living in environments with worsened infrastructure experienced worse health at baseline (−0.77; 95% CI: −1.53, −0.01). Respondents who experienced worsened environmental pollution had the worst PCS (−1.21; 95% CI: −2.11, −0.31), but stable moderate (−1.04; 95% CI: −1.56, −0.51) and worst pollution (−0.72; 95% CI: −1.29, −0.14) were also related to worse PCS. Living under stable worst (−0.97; 95% CI: −1.54, −0.39) and worsened (−1.00; 95% CI: −1.75, −0.24) housing conditions was connected to lower PCS score at baseline as well.

**Table 2:** Associations between changes in living environment before baseline and physical component summary (PCS) at baseline (Level Model^a^) as well as changes in PCS from baseline onwards (Change Model^b^), German Socio-Economic Panel 1999–2014

Variable	Level model^c^		Change model^d^		Change model with interaction^d^	
	Coeff.	95% CI	Coeff.	95% CI	Coeff.	95% CI
Infrastructure						
Stable best	Ref.		Ref.			
Stable moderate	−0.44	−1.00, 0.12	−0.14	−0.61, 0.32		
Stable worst	−0.56	−1.13, 0.01	−0.84	−1.33, −0.35		
Improved	−0.03	−0.99, 0.93	−0.49	−1.35, 0.36		
Worsened	−0.77	−1.53, −0.01	−0.37	−1.00, 0.25		
Environmental pollution						
Stable best	Ref.		Ref.		Ref.	
Stable moderate	−1.04	−1.56, −0.51	−0.75	−1.18, −0.31	−0.85	−1.46, −0.25
Stable worst	−0.72	−1.29, −0.14	−0.66	−1.15, −0.17	−0.97	−1.64, −0.31
Improved	0.15	−0.66, 0.96	−0.53	−1.23, 0.17	−0.25	−1.21, 0.71
Worsened	−1.21	−2.11, −0.31	−0.86	−1.64, −0.08	−1.73	−2.86, −0.60
Housing conditions						
Stable good	Ref.		Ref.			
Stable in need of renovation	−0.97	−1.54, −0.39	−0.28	−0.76, 0.20		
Improved	−0.17	−0.86, 0.51	−0.08	−0.64, 0.49		
Worsened	−1.00	−1.75, −0.24	−0.55	−1.17, 0.09		
Environmental pollution × sex						
Stable moderate, women					0.20	−0.65, 1.05
Stable worst, women					0.57	−0.34, 1.48
Improved, women					−0.56	−1.93, 0.81
Worsened, women					1.67	0.15, 3.19
Sex						
Men					Ref.	
Women					−0.42	−1.01, 0.18

Notes: Coeff., coefficient; CI, confidence interval; Ref., reference.

a Estimated from a linear regression with robust standard errors by Huber/White.

b Estimated from generalized estimating equations using the identity link function and a normally distributed outcome variable.

c Model was controlled for time-invariant characteristics at baseline (age, remoteness, education, marital status, nutrition behaviour, year of baseline, GSOEP-subsample) and time-varying characteristics up to baseline (weekly working hours, household income, subjective health, smoking).

d Model was controlled for all variables from the Level Model (see footnote c) and additionally for PCS at baseline as well as time-varying characteristics from baseline onwards (start or stop smoking, transition to unemployment or retirement, changing marital status, death of the partner, distance between follow-ups and baseline in years).

For all characteristics we found that PCS of people who experienced improved conditions did not differ significantly from those with stable best conditions.

### Change Model

For infrastructure, stable worst (−0.84; 95% CI: −1.33, −0.35) conditions were associated with negative changes in PCS. For environmental pollution, living under stable moderate (−0.75; 95% CI: −1.18, −0.31), worst (−0.66; 95% CI: −1.15, −0.17) and worsened (−0.86; 95% CI: −1.64, −0.08) conditions was connected with negative health changes. Again, changes in the PCS of respondents with improved conditions did not differ significantly from those with stable best conditions.

**Figure 2:**
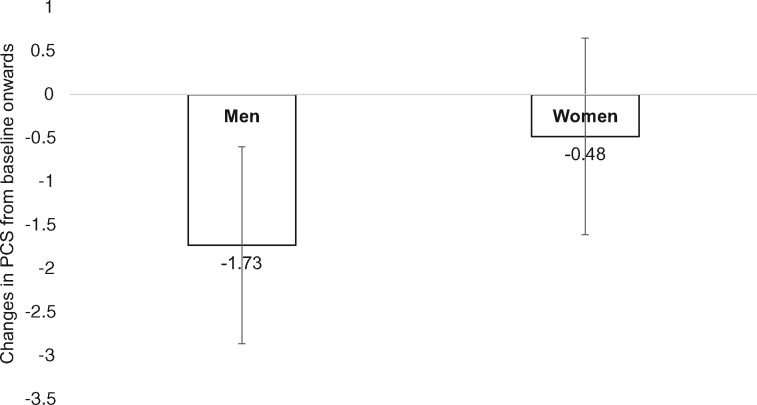
Interaction between sex and worsened environmental pollution by using the Change Model (ref. men, stable best environmental pollution)

In addition, we found an interaction between environmental pollution and sex in the Change Model, which indicates that men were more prone to worsened pollution (−1.73; 95% CI: −2.86, −0.60) than women (−0.48; 95% CI: −1.54, 0.58) (figure 2, table 2).

To strengthen the causality of the Change Model, we only explored non-householders, who did not report perceived living environment by themselves and used the reports of another member of the household. These models were estimated sex-specific (Supplementary table S4). Results did not change and underlined the sex differences concerning environmental pollution.

## Discussion

### Summary of principal findings

Stable moderate and worst as well as worsened environmental pollution and infrastructure were associated with worse PCS at baseline, which was also true for stable poor and worsened housing conditions. Stable worst infrastructure was associated with negative changes in PCS for both sexes. Men’s changes in PCS were more affected by worsened environmental pollution than women’s.

### Evaluation of data and methods

Our study has two strengths compared with previous studies in the field. First, we considered both repeated health and living environment assessments, which had only been done by a few previous studies in the field.^7^ To the best of our knowledge, this is the first study that has explored changes in health over time among non-movers, and not only health levels, while additionally controlling for time-varying individual characteristics. We controlled for baseline health to make sure that the results were not confounded by poor or good health at baseline.

Second, our results stem from a study design which imposes a strict time dimension between exposure and outcome to avoid reverse causation, and we concentrated on all-time non-movers (before and after baseline) to exclude positive health selection into living environments due to relocation. Investigating movers is problematic because of either unobserved individual characteristics of the movers or the health status as a reason for an individual’s decision to move.^3^^,^^4^^,^^12^

Nevertheless, our study does have some limitations. First, the design covers short-term changes in living environment, i.e. changes within 5 years. Contextual effects may, however, show effects over the entire life course in the form of cumulated exposures or in critical periods.^21^ However, for air pollution it has been shown that even short-term deprivations influence people’s health.^22^^,^^23^ Due to their proximity to physical health it is especially the changes in physical environment, represented in our study by environmental pollution and infrastructure, which might become health-relevant rather rapidly.

Second, perceived living environment in the GSOEP was assessed at the household level. Even if there is a certain degree of autocorrelation between the household members within a household, perception can differ among the individual household members. However, it is unlikely that our gender-specific findings are the result of a gender bias in asking household heads only, as the distribution is 57.16% male and 42.84% female.

Third, the living environment measures used stem from householders’ subjective assessments. Using both subjective outcome measures and subjective predictors can lead to potential same-source bias.^24^^,^^25^

However, the causal explanation of our findings is strengthened by a series of (sensitivity) analyses, which takes care of some of the limitations and leads to unchanged results. First, we restricted the sample to non-householders who do not suffer from same-source bias (Supplementary table S4). Second, we estimated a Change Model with at least two health changes for each individual assuming that one health change might be potentially unreliable (Supplementary table S5). Third, we estimated a Level Model including all participants with at least one health measurement at baseline (Supplementary figure S3) to tackle a possible selection bias (Supplementary table S6).

### Interpretation of findings

Our study shows that, in line with our hypotheses, stable suboptimal and declining levels of environmental pollution and infrastructure influence the current level of health as well as changes in health.

On the one hand, this result suggests that suboptimal conditions have short- and long-term negative effects; on the other hand, observing a relationship for changes in health strengthens the causal interpretation of our findings. For housing conditions, we did not find relations in the Change Model, suggesting that these conditions have a predominantly short-term effect on physical health only. Furthermore, including the Change Model makes it possible to compare the results of the strategy commonly used in the field (using health levels) and our novel strategy used in this study (using changes in health over time). The commonalities and differences between the findings in the Level Model and the Change Model point to the importance of both approaches. There was also strong evidence for sex-specific relationships because men’s changes in physical health were more affected by worsened environmental pollution than women’s.

One major mechanism behind the observed short- and long-term relationships might be that beneficial or deprived physical characteristics of living environments influence people’s bodily conditions and may delay or accelerate ageing processes in addition to individual age-related factors.^26^ A previous longitudinal study,^27^ which focussed on changes in the built environment and changes in amount of walking, found that an increasing density of infrastructure promotes more walking. Walking provides better health^28^ due to positive effects on physical and cognitive functioning.^29^ There is also empirical evidence that higher levels of environmental pollution, e.g. air and noise pollution, are associated with worse physical and mental health. Exposures to fine particles impair the lung function and cause further physical and cognitive decline thereafter.^30^ It has also been shown that relocating from high to low polluted areas (or vice versa) is associated with subsequent changes in lung function growth.^31^A high level of noise pollution, especially nocturnal noise exposure, influences people’s sleeping behaviour and can thus affect health negatively.^32^

We only found associations for housing conditions in the Level Model. This could be explained by two possible mechanisms: First, housing conditions only have a short-term (and not a long-term) effect on physical health. Second, changes in housing conditions reflect migration trajectories of the past and have no causal effect on physical health. However, another previous study on changes in housing conditions on health gives some support to the hypothesis that changes in housing conditions do indeed have a short-term effect on physical health outcomes.^33^

Our sex-specific finding, that worsened environmental pollution and changes in physical health were more negative for men’s health developments, is supported by a previous cross-sectional study which found associations between perceived physical problems (air quality, waste disposal) and self-rated health only for men.^34^ Three possible explanations for gender differences in the association between changes in the living environment and health are discussed in the literature.^35^

First, men and women perceive or experience their living environments in different ways.^36^ In our study, this hypothesis is less applicable, because the questions on the living environment were answered by the key-person of the households only.

Second, the dose of exposure to the different living environmental characteristics differ between men and women, which may also be influenced by different social roles.^37^ Results from the German Time Use Survey in 2012/13^38^ seem to support this explanation. That is, men spend more time with outside physical activities.

Third, sex differences in the vulnerability for specific (changes in) environmental characteristics, in terms of sensibility of bodies and biological systems,^39^ can lead to different health consequences for men than for women.

To summarize, our study reinforces existing theoretical frameworks and shows that not only lifestyle but also the external characteristics of living environment affects people’s health.^40^

## Conclusion

The present findings provide strong evidence that people’s perceived physical health depends, among other things, on their housing conditions, as well as the quality of the infrastructure and the environmental pollution they experience in their immediate surroundings. A suboptimal living environment has short- and long-term negative effects on physical health. Because even short-term changes in the living environment have an immediate influence on an individual’s health status and health trajectories, public attention to living environment is essential in fighting existing health inequalities.

## Supplementary Material

ckz044_Supplementary_Materials
