# Supplementary material for: Effects of changes in living environment on physical health: a prospective German cohort study of non-movers
Source: Eur J Public Health. 2019 Mar 18;29(6):1147–53. doi: 10.1093/eurpub/ckz044 (PMC6896981; doi:10.1093/eurpub/ckz044)
Supplement: ckz044_Supplementary_Materials [file ckz044_supplementary_materials.zip › ckz044-suppl_data/Supplementary Table 4.docx]

**Supplementary Table 4**. Change Model^a^ - Associations between infrastructure, environmental pollution, housing conditions and changes in Physical Component Summary (PCS) only for non-householders, German Socio-Economic Panel 1999-2014

|  | **Non-householders** | | | |
| --- | --- | --- | --- | --- |
| **Variable** | **Men^b^ (n = 555)** | | **Women^b^ (n = 1,357)** | |
|  | **Coeff.** | **95% CI** | **Coeff.** | **95% CI** |
| Infrastructure |  |  |  |  |
| Stable best | Ref. |  | Ref. |  |
| Stable moderate | 0.03 | -1.21, 1.29 | -0.46 | -1.32, 0.41 |
| Stable worst | -1.69 | -3.03, -0.35 | -0.99 | -1.84, -0.14 |
| Improved | -0.51 | -2.33, 1.31 | 0.14 | -1.54, 1.81 |
| Worsened | -1.53 | -3.26, 0.20 | 0.07 | -1.11, 1.25 |
| Environmental pollution |  |  |  |  |
| Stable best | Ref. |  | Ref. |  |
| Stable moderate | -1.60 | -2.75, -0.45 | -0.80 | -1.61, 0.02 |
| Stable worst | -1.28 | -2.51, -0.04 | -0.62 | -1.58, 0.33 |
| Improved | -1.46 | -3.45, 0.53 | -0.47 | -1.76, 0.81 |
| Worsened | -2.87 | -4.83, -0,91 | 0.59 | -0.74, 1.91 |
| Housing conditions |  |  |  |  |
| Stable best | Ref. |  | Ref. |  |
| Stable worst | 0.72 | -0.60, 2.05 | -0.12 | -1.01, 0.77 |
| Improved | 0.77 | -0.82, 2.36 | -0.35 | -1.49, 0.78 |
| Worsened | 1.07 | -0.41, 2.55 | -0.10 | -1.30, 1.09 |

Coeff., coefficient; CI, confidence interval; Ref., reference.

^a^ Estimated from generalised estimating equations using the identity link function and a normally distributed outcome variable.

^b^ Model was controlled for time-invariant characteristics at baseline (age, remoteness, education, marital status, nutrition behaviour, year of baseline, GSOEP-subsample), time-varying characteristics up to baseline (weekly working hours, household income, subjective health, smoking) and additionally for PCS at baseline as well as time-varying characteristics from baseline onwards (start or stop smoking, transition to unemployment or retirement, changing marital status, death of the partner, distance between follow-ups and baseline in years).
