# Supplementary material for: Effects of changes in living environment on physical health: a prospective German cohort study of non-movers
Source: Eur J Public Health. 2019 Mar 18;29(6):1147–53. doi: 10.1093/eurpub/ckz044 (PMC6896981; doi:10.1093/eurpub/ckz044)
Supplement: ckz044_Supplementary_Materials [file ckz044_supplementary_materials.zip › ckz044-suppl_data/Supplementary Table 5.docx]

| **Variable** | **Change Model**^b^ | |  | **Change Model with interaction**^b^ | |
| --- | --- | --- | --- | --- | --- |
|  | **Coeff.** | **95% CI** |  | **Coeff.** | **95% CI** |
| Infrastructure |  |  |  |  |  |
| Stable best | Ref. |  |  |  |  |
| Stable moderate | -0.18 | -0.67, 0.31 |  |  |  |
| Stable worst | -0.99 | -1.51, -0.47 |  |  |  |
| Improved | -0.56 | -1.47, 0.34 |  |  |  |
| Worsened | -0.48 | -1.15, 0.19 |  |  |  |
| Environmental pollution |  |  |  |  |  |
| Stable best | Ref. |  |  | Ref. |  |
| Stable moderate | -0.82 | -1.28, -0.36 |  | -0.90 | -1.54, -0.26 |
| Stable worst | -0.70 | -1.22, -0.18 |  | -1.01 | -1.72, -0.31 |
| Improved | -0.53 | -1.28, 0.22 |  | -0.32 | -1.35, 0.70 |
| Worsened | -0.91 | -1.75, -0.07 |  | -1.96 | -3.18, -0.74 |
| Housing conditions |  |  |  |  |  |
| Stable good | Ref. |  |  |  |  |
| Stable in need of renovation | -0.31 | -0.82, 0.21 |  |  |  |
| Improved | 0.01 | -0.59, 0.61 |  |  |  |
| Worsened | -0.56 | -1.24, 0.13 |  |  |  |
| Environmental pollution x sex |  |  |  |  |  |
| Stable moderate, women |  |  |  | 0.14 | -0.76, 1.03 |
| Stable worst, women |  |  |  | 0.56 | -0.40, 1.53 |
| Improved, women |  |  |  | -0.42 | -1.88, 1.04 |
| Worsened, women |  |  |  | 2.02 | 0.38, 3.66 |
| Sex |  |  |  |  |  |
| Men |  |  |  | Ref. |  |
| Women |  |  |  | -0.43 | -1.06, 0.19 |

**Supplementary Table 5.** Change Model^a^ **-** Associations between changes in living environment before baseline and changes in Physical Component Summary from baseline onwards including participants with at least two health changes (n = 3,779), German Socio Economic Panel 1999-2014

Coeff., coefficient; CI, confidence interval; Ref., reference.

^a^ Estimated from generalised estimating equations using the identity link function and a normally distributed outcome variable.

^b^ Model was controlled for time-invariant characteristics at baseline (age, remoteness, education, marital status, nutrition behaviour, year of baseline, GSOEP-subsample), time-varying characteristics up to baseline (weekly working hours, household income, subjective health, smoking) and additionally for PCS at baseline as well as time-varying characteristics from baseline onwards (start or stop smoking, transition to unemployment or retirement, changing marital status, death of the partner, distance between follow-ups and baseline in years.
