# Supplementary material for: Effects of changes in living environment on physical health: a prospective German cohort study of non-movers
Source: Eur J Public Health. 2019 Mar 18;29(6):1147–53. doi: 10.1093/eurpub/ckz044 (PMC6896981; doi:10.1093/eurpub/ckz044)
Supplement: ckz044_Supplementary_Materials [file ckz044_supplementary_materials.zip › ckz044-suppl_data/Supplementary Table 6.docx]

**Supplementary Table 6.** Level Model^a^ **-** Associations between changes in living environment before baseline and Physical Component Summary (PCS) at baseline including all participants with at least one health measurement at baseline (n = 5,748), German Socio-Economic Panel 1999-2014

| **Variable** | **Level Model**^b^ | |
| --- | --- | --- |
|  | **Coeff.** | **95% CI** |
| Infrastructure |  |  |
| Stable best | Ref. |  |
| Stable moderate | -0.39 | -0.89, 0.12 |
| Stable worst | -0.49 | -1.00, 0.02 |
| Improved | -0.04 | -0.85, 0.78 |
| Worsened | -0.74 | -1.43, -0.06 |
| Environmental pollution |  |  |
| Stable best | Ref. |  |
| Stable moderate | -0.88 | -1.34, -0.41 |
| Stable worst | -0.70 | -1.21, -0.19 |
| Improved | -0.06 | -0.79, 0.68 |
| Worsened | -1.19 | -1.96, -0.42 |
| Housing conditions |  |  |
| Stable good | Ref. |  |
| Stable in need of renovation | -0.90 | -1.40, -0.40 |
| Improved | -0.43 | -1.05, 0.18 |
| Worsened | -0.84 | -1.45, -0,22 |

Coeff., coefficient; CI, confidence interval; Ref., reference.

^a^ Estimated from a linear regression with robust standard errors by Huber/White.

^b^ Model was controlled for time-invariant characteristics at baseline (age, remoteness, education, marital status, nutrition behaviour, year of baseline, GSOEP-subsample) and time-varying characteristics up to baseline (weekly working hours, household income, subjective health, smoking).
