# Supplementary material for: Effects of changes in living environment on physical health: a prospective German cohort study of non-movers
Source: Eur J Public Health. 2019 Mar 18;29(6):1147–53. doi: 10.1093/eurpub/ckz044 (PMC6896981; doi:10.1093/eurpub/ckz044)
Supplement: ckz044_Supplementary_Materials [file ckz044_supplementary_materials.zip › ckz044-suppl_data/Supplementary Table 3.docx]

**Supplementary Table 3.** Descriptive statistics of the analysis sample (n = 4,601, non-movers) for all variables used, German Socio-Economic Panel 1999-2014

| **Variable** | **%** | **No. of obs.** | **Mean^a^**  **(SD)** |
| --- | --- | --- | --- |
| PCS at baseline |  | 4,601 | 47.43  (10.0) |
| PCS from baseline onwards |  | 16,076 | 46.50  (10.1) |
| Age |  | 4,601 | 54.56  (14.5) |
| Infrastructure |  |  |  |
| Stable best | 27.49 | 1,265 |  |
| Stable moderate | 27.65 | 1,272 |  |
| Stable worst | 29.21 | 1,344 |  |
| Improved | 5.85 | 269 |  |
| Worsened | 9.80 | 451 |  |
| Environmental pollution |  |  |  |
| Stable best | 1,723 | 37.45 |  |
| Stable moderate | 1,173 | 25.49 |  |
| Stable worst | 1,019 | 22.15 |  |
| Improved | 413 | 8.98 |  |
| Worsened | 273 | 5.93 |  |
| Housing conditions |  |  |  |
| Stable good | 2,905 | 63.14 |  |
| Stable in need of renovation | 823 | 17.89 |  |
| Improved | 479 | 10.41 |  |
| Worsened | 394 | 8.56 |  |
| Remoteness |  |  |  |
| Residence in the city centre | 393 | 8.54 |  |
| Distance < 10 kilometres | 1,026 | 22.30 |  |
| Distance 10-24 kilometres | 1,233 | 26.80 |  |
| Distance 25-39 kilometres | 754 | 16.39 |  |
| Distance 40-59 kilometres | 642 | 13.95 |  |
| Distance > 59 kilometres | 553 | 12.02 |  |
| Education |  |  |  |
| Low | 828 | 18.00 |  |
| Middle | 2,457 | 53.40 |  |
| High | 1,316 | 28.60 |  |
| Weekly working hours |  |  |  |
| Stable full-time employment | 1,494 | 32.47 |  |
| Stable part-time employment | 275 | 5.98 |  |
| Stable marginal employment | 54 | 1.17 |  |
| Stable not employed/retired | 1,712 | 37.21 |  |
| Increased working hours | 380 | 8.26 |  |
| Decreased working hours | 686 | 14.91 |  |
| Household income |  |  |  |
| Stable 1. quintile | 590 | 12.82 |  |
| Stable 2. quintile | 481 | 10.45 |  |
| Stable 3. quintile | 364 | 7.91 |  |
| Stable 4. quintile | 286 | 6.22 |  |
| Stable 5. quintile | 450 | 9.78 |  |
| Increased income | 1,677 | 36.45 |  |
| Decreased income | 753 | 16.37 |  |
| Subjective health |  |  |  |
| Stable very good | 97 | 2.11 |  |
| Stable good | 1,061 | 23.06 |  |
| Stable satisfactory | 980 | 21.30 |  |
| Stable poor | 272 | 5.91 |  |
| Stable bad | 59 | 1.28 |  |
| Improved | 845 | 18.37 |  |
| Worsened | 1,287 | 27.97 |  |
| Smoking |  |  |  |
| Yes | 987 | 21.45 |  |
| No | 3,208 | 69.72 |  |
| Started | 259 | 5.63 |  |
| Stopped | 147 | 3.19 |  |
| Marital status |  |  |  |
| Married | 3,381 | 73.48 |  |
| Single | 506 | 11.00 |  |
| Widowed | 430 | 9.35 |  |
| Divorced | 248 | 5.39 |  |
| Separated | 36 | 0.78 |  |
| Nutrition behaviour |  |  |  |
| Very much | 421 | 9.15 |  |
| Much | 2,097 | 45.58 |  |
| Not so much | 1,854 | 40.30 |  |
| Not at all | 229 | 4.98 |  |
| Events after baseline |  |  |  |
| Start smoking | 191 | 1.66 |  |
| Stop smoking | 384 | 3.35 |  |
| Unemployment/retirement | 322 | 2.81 |  |
| Separated | 54 | 0.47 |  |
| Divorced | 36 | 0.31 |  |
| Married | 35 | 0.31 |  |
| Death of the partner | 59 | 0.51 |  |

No., number; Obs., observations; SD, standard deviation; PCS, Physical Component Summary.

^a^ Mean PCS at baseline was calculated by using the measurement of PCS at baseline and mean PCS from baseline onwards was calculated by using the multiple PCS measurements from baseline onwards.
